# Prophylaxis of ibuprofen in acute mountain sickness: A protocol for systematic review and meta-analysis

**DOI:** 10.1097/MD.0000000000023233

**Published:** 2020-11-13

**Authors:** Hai Yi, Kuiying Wang, Xinyu Gan, Li Li, Qian Zhang, Jiao Xiang, Xiuwei Yuan, Yugang Zhang, Yonghua Wang

**Affiliations:** a Department of Hematology; b Department of Wounded Management; c Department of Transfusion Medicine; d Department of Experimental Medicine; e Department of Infectious Diseases; f Outpatient Department; g Department of Health Service; h Department of Nursing, the General Hospital of Western Theater Command, PLA, Chengdu, China.

**Keywords:** acute mountain sickness, ibuprofen, prophylaxis, systematic review, meta-analysis

## Abstract

**Background:**

Acute mountain sickness (AMS) is the effect when people accessing high altitude in a short period of time. As a cyclooxygenase (COX) inhibitor, ibuprofen could alleviate the symptoms of AMS. However, whether it can prevent AMS or not is still controversial. It is necessary to perform a meta-analysis to evaluate the role of ibuprofen in AMS prophylaxis.

**Methods:**

PubMed, EMBASE, Medline, ISI Web of Science, Cochrane Library, China National Knowledge Infrastructure (CNKI) will be searched for the relevant published studies that explored the value of ibuprofen in AMS prophylaxis from inception to October 2020. The data will be independently extracted by 2 researchers. Risk of bias will be evaluated based on Cochrane risk of bias assessment tool. Heterogeneity among the included studies will be evaluated by χ^2^ and *I*^2^ values. The meta-analysis was conducted by RevMan software version 5.3.

**Results:**

This study will evaluate the role of ibuprofen in AMS prophylaxis.

**Conclusion:**

This study will summarize the current evidence of ibuprofen in AMS prophylaxis, which could further guide the recommendation in prevention of AMS.

Open Science Framework (OSF) registration number: October 8, 2020. osf.io/n3mjt.

## 1 Background

Acute mountain sickness (AMS) is the effect when people accessing high altitude (>2500 meters) in a short period of time, which is characterized by a group of symptoms such as headache, fatigue, shortness of breath, dizziness, nausea and vomiting, diarrhea, disturbed sleep, et al.^[1,2]^ Also, it might progress to encephaledema or pneumonedema,^[3,4]^ which are life-threatening medical conditions. The pathogenesis of AMS is not well understood. Until now, limited drugs are recommended to AMS prophylaxis.^[5–7]^ Acetazolamide, which is a carbonic anhydrase inhibitor, has been proven to prevent AMS in a large number of studies.^[8,9]^ Due to the certain side effects such as gastrointestinal reactions, abnormal urination and electrolyte disorders,^[10–12]^ it is not suitable for certain people. Dexamethasone, which is a long-acting glucocorticoid, can also prevent and alleviate the symptoms of AMS.^[13]^ But it is not suitable for people with hypertension or diabetes mellitus.^[14]^ New therapeutic agents are urgently needed.

Recently, emerging data showed that hypoxia was associated with inflammation.^[15,16]^ Also our data shows that AMS is related to elavated inflammatory cytokines (Hai Yi, MD, unpublished data, October 2020), indicate that inflammation might mediate the pathophysiology of AMS. When expose to hypoxia, the arachidonic acid (AA) metabolism pathway was excessively enhanced.^[17]^ Cyclooxygenase (COX) inhibitor ibuprofen could effectively target AA pathway and alleviate the symptoms of AMS.^[15,18,19]^ However, whether it can prevent AMS or not is still controversial.^[20]^ It is necessary to perform a meta-analysis to evaluate the role of ibuprofen in AMS prophylaxis.

## 2 Methods

### 2.1 Study registration

The protocol of the systematic review has been registered. Registration: OSF registration. October 8, 2020. URL: https://osf.io/n3mjt. It has been reported following the guideline of Preferred Reporting Items for Systematic Reviews and Meta-Analysis Protocol statement.

### 2.2 Ethics

This study is a systematic review; the outcomes are based on the published evidence, so examination and agreement by the ethics committee are not required. We plan to publish the results in a journal or conference presentation.

### 2.3 Eligibility criteria

#### 2.3.1 Type of study

This review will include randomized controlled trials of ibuprofen for AMS prevention. Language is limited in English and Chinese. Non-RCTs, observational studies, case reports, crossover studies and laboratory studies will be excluded.

#### 2.3.2 Participants

We will include participants of 18 years or older, of any sex and ethnicity. All the participants following rapid ascent (in 3 days) to a high altitude (>2500 meters) were assessed the systems of AMS, according to the Lake Louise criteria.^[21]^ The participants who had underlying diseases or complications would be excluded.

#### 2.3.3 Interventions

The experiment group use ibuprofen, with no limit of dose and frequency. Any other drug could not be used. The control group use other medicine, placebo or none.

#### 2.3.4 Outcome measurements

Our primary objective was to assess the effect of ibuprofen in AMS prophylaxis. The symptoms of AMS were assessed using the Lake Louise criteria^[21]^ or Alternative methods,^[22]^ such as the Environmental Symptoms Questionnaire, Symptom Questionnaire of AMS, General High-Altitude Questionnaire (GHAQ), or clinical examination, for instance, peripheral oxygen saturation.

### 2.4 Data sources and search strategy

A literature search will be performed in PubMed, Medline, EMBASE, Cochrane Library, Web of Science and CNKI from their inception to October 10, 2020. We will limit our search in English and Chinese. The search strategy of Medline was shown in Table 1. Other electronic databases will be used by the similar retrieval strategy.

**Table 1:** Search strategy applied in MEDLINE database.

Number	Search terms
1	acute mountain sickness
2	acute mountain illness
3	acute mountain headache
4	altitude headache
5	altitude sickness
6	high altitude cerebral edema
7	high altitude pulmonary edema
8	encephaledema
9	pneumonedema
10	or 1–9
11	ibuprofen
12	randomized controlled trial
13	controlled clinical trial
14	single blind
15	double blind
16	clinial trials
17	RCT
18	or 12–17
19	10 and 11 and 18

### 2.5 Data collection and analysis

#### 2.5.1 Studies selection

**Figure 1:**
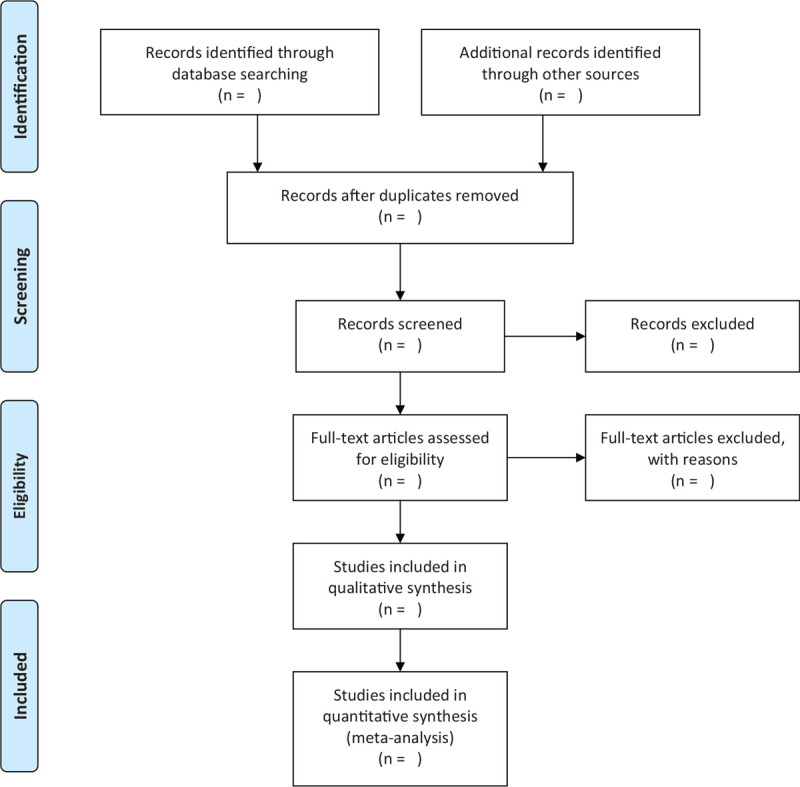
Flow chart of the study selection. This figure shows the Identification, Screening, Eligibility and Included when we searching articles.

Two reviewers (HY and KW) will preliminarily screen the titles and abstracts independently. Then, the full text of the relavant studies will be downloaded for further selection according to the inclusion criteria. Any disagreements will be discussed and agreement will be reached. However, if a consensus can not be made, a third researcher (XG) will make the final decision. The selection process is displayed in the PRISMA flow chart (Figure 1).

#### 2.5.2 Data extraction and management

Two reviewers will extract the data independently using a standardized excel form and confirm by a third researcher. The following items will be extracted: first author, year of publication, regions, sample size, sample types, year of data collection, ages, genders and ethnicity of participants, intervention of control group, dose and frequency of intervention, adverse effects. If there is missing information, we will contact the corresponding author for more details.

#### 2.5.3 Assessment of risk of bias in included studies

Two reviewers will assess the risk of bias of included studies independently by using the Cochrane risk of bias assessment tool.^[23]^ The following items, random sequence generation, allocation concealment, blinding of participants and personnel, blinding of outcome assessment, incomplete outcome data, selective reporting, and other bias, are judged to be low-risk grade, high-risk grade and unclear grade. Two reviewers will assess the risk of bias independently and any disagreements between the 2 reviewers will be resolved by a discussion of all reviewers.

#### 2.5.4 Dealing with missing data

If the relevant data in the study is incomplete, the reviews will contact the corresponding author via email for more information. If the missing data cannot be obtained, sensitivity analysis will be conducted to evaluate the impact of missing data on the conclusions of the study.

#### 2.5.5 Data analysis

The RevMan 5.3 software (The Cochrane Collaboration) was used for statistical analysis. The heterogeneity was determined by χ^2^ and *I*^*2*^ values. If there is no heterogeneity (*I*^2^ < 50%, *P* > .05), the data are synthesized by fixed-effects model; otherwise, the random-effects model would be considered.^[13]^ An unadjusted odds ratios (OR) with 95% confidence intervals (CI) was used to evaluate the effect between the 2 groups.

#### 2.5.6 Subgroup analysis

If the results of the studies are heterogeneous, a subgroup analysis will be conducted to investigate the differences in age, gender, race, altitude, dose and frequency of ibuprofen, et al.

#### 2.5.7 Sensitivity analysis

In order to determine the stability of study finding, sensitivity analysis will be performed.

#### 2.5.8 Publication bias assessment

If no few than 10 studies were included, funnel plots will be used to assess publication bias.^[13]^

#### 2.5.9 Grading the quality of evidence

The Grading of Recommendations Assessment, Development, and Evaluation (GRADE) will be used to assess the quality of evidence. The quality of evidence will be indicated as high, moderate, low, and very low. Disagreements between the 2 reviewers will be resolved by a discussion of all reviewers during the quality grading.

## 3 Discussion

The incidence of AMS is relatively high, from 23.9% to 53%,^[24–26]^ in people accessing high altitude in a short period of time. Also, high altitude encephaledema or pneumonedema, which is a potentially fatal condition, is seen in 0.1% to 10% of people with AMS.^[27]^ That situation has a high mortality if untreated. For people accessing high altitude, prevention of AMS can alleviate the symptoms and improve performance of climbers, which has great social significance. Acetazolamide and dexamethasone are the only 2 drugs that are currently recommended for the prevention of AMS.^[8,13]^ As a COX inhibitor, ibuprofen can effectively prevent AMS according to some literature.^[18,19]^ However, negative results also been made in some studies.^[20,28]^ So ibuprofen has not been recommended so far. It is important to clarify the effect of ibuprofen in AMS prevention.

This systematic review from randomised-controlled trials might give a detail analysis evaluating the use of ibuprofen in AMS prophylaxis. Also, we will explore the best dosage and course of ibuprofen. The findings of this review will be widely disseminated through peer-reviewed journal and conference presentation. The conclusion of this review will provide valuable evidence of ibuprofen for routine practice to prevent AMS.

## Author contributions

**Conceptualization:** Xiuwei Yuan, Yugang Zhang, Yonghua Wang.

**Funding acquisition:** Hai Yi.

**Methodology:** Hai Yi, Kuiying Wang, Xinyu Gan, Li Li, Qian Zhang, Jiao Xiang.

**Project administration:** Xiuwei Yuan, Yugang Zhang, Yonghua Wang.

**Writing – original draft:** Hai Yi.

**Writing – review & editing:** Yonghua Wang.

## References

[1] Garrido E Botella de Maglia J Castillo O Acute, subacute and chronic mountain sickness. Rev Clin Esp 2020;17:S0014-2565(20)30064-3. 10.1016/j.rceng.2019.12.009 34583826

[2] Li Y Zhang Y Zhang Y Research advances in pathogenesis and prophylactic measures of acute high altitude illness. Resp Med 2018;145:145–52. 10.1016/j.rmed.2018.11.004 30509704

[3] Dawadi S Basnyat B Adhikari S A review of medical problems in himalayan porters. High Alt Med Biol 2020;21:109–13. 32311284 10.1089/ham.2020.0004

[4] Richalet JP Larmignat P Poignard P Transient cerebral ischemia at high altitude and hyper-responsiveness to hypoxia. High Alt Med Biol 2020;21:105–8. 31971870 10.1089/ham.2019.0100

[5] Metrailler P Greiser J Dietrich G Swiss mountain guides: medical education, knowledge, and practice. High Alt Med Biol 2019;20:251–61. 31373841 10.1089/ham.2018.0124

[6] Joyce KE Lucas SJE Imray CHE Advances in the available non-biological pharmacotherapy prevention and treatment of acute mountain sickness and high altitude cerebral and pulmonary oedema. Expert Opin Pharmacother 2018;19:1891–902. 30307756 10.1080/14656566.2018.1528228

[7] Simancas-Racines D Arevalo-Rodriguez I Osorio D Interventions for treating acute high altitude illness. Cochrane Database Syst Rev 2018;6:CD009567. 29959871 10.1002/14651858.CD009567.pub2 PMC6513207

[8] Tapia L Irarrazaval S Acetazolamide for the treatment of acute mountain sickness. Medwave 2019;19:e7737. 31891352 10.5867/medwave.2019.11.7736

[9] Lipman GS Jurkiewicz C Winstead-Derlega C Day of Ascent Dosing of Acetazolamide for Prevention of Acute Mountain Sickness. High Alt Med Biol 2019;20:271–8. 31259608 10.1089/ham.2019.0007

[10] Collier DJ Wolff CB Hedges AM Benzolamide improves oxygenation and reduces acute mountain sickness during a high-altitude trek and has fewer side effects than acetazolamide at sea level. Pharmacol Res Perspect 2016;4:e00203. 27433337 10.1002/prp2.203 PMC4876137

[11] Swenson ER Pharmacology of acute mountain sickness: old drugs and newer thinking. J Appl Physiol 2016;120:204–15. 26294748 10.1152/japplphysiol.00443.2015

[12] Pun M Side-effect of acetazolamide in prevention of acute mountain sickness. S Afr Med J 2012;102 (3 Pt 1):114 author reply 114. 22380891 10.7196/samj.5348

[13] Tang E Chen Y Luo Y Dexamethasone for the prevention of acute mountain sickness: systematic review and meta-analysis. Int J Cardiol 2014;173:133–8. 24679688 10.1016/j.ijcard.2014.03.019

[14] Polderman JAW Farhang-Razi V van Dieren S Adverse side-effects of dexamethasone in surgical patients - an abridged Cochrane systematic review. Anaesthesia 2019;74:929–39. 30821852 10.1111/anae.14610

[15] Chauhan G Roy K Kumar G Distinct influence of COX-1 and COX-2 on neuroinflammatory response and associated cognitive deficits during high altitude hypoxia. Neuropharmacology 2019;146:138–48. 30476507 10.1016/j.neuropharm.2018.11.026

[16] Zhou Y Huang X Zhao T Hypoxia augments LPS-induced inflammation and triggers high altitude cerebral edema in mice. Brain Behav Immun 2017;64:266–75. 28433745 10.1016/j.bbi.2017.04.013

[17] Liu C Liu B Liu L Arachidonic acid metabolism pathway is not only dominant in metabolic modulation but associated with phenotypic variation after acute hypoxia exposure. Frontiers Physiol 2018;9:236. 10.3389/fphys.2018.00236 PMC5864929 29615930

[18] Burns P Lipman GS Warner K Altitude sickness prevention with ibuprofen relative to acetazolamide. Am J Med 2019;132:247–51. 30419226 10.1016/j.amjmed.2018.10.021

[19] Kanaan NC Peterson AL Pun M Prophylactic acetaminophen or ibuprofen results in equivalent acute mountain sickness incidence at high altitude: a prospective randomized trial. Wilderness Environ Med 2017;28:72–8. 28479001 10.1016/j.wem.2016.12.011

[20] Lundeberg J Feiner JR Schober A Increased cytokines at high altitude: lack of effect of ibuprofen on acute mountain sickness, physiological variables, or cytokine levels. High Alt Med Biol 2018;19:249–58. 29924642 10.1089/ham.2017.0144

[21] Vann RD Pollock NW Pieper CF Statistical models of acute mountain sickness. High Alt Med Biol 2005;6:32–42. Spring. 15772498 10.1089/ham.2005.6.32

[22] Wagner DR Teramoto M Knott JR Comparison of scoring systems for assessment of acute mountain sickness. High Alt Med Biol 2012;13:245–51. 23270440 10.1089/ham.2012.1030

[23] Higgins JP Altman DG Gotzsche PC The Cochrane Collaboration's tool for assessing risk of bias in randomised trials. BMJ 2011;343:d5928. 22008217 10.1136/bmj.d5928 PMC3196245

[24] Hackett PH Rennie D Levine HD The incidence, importance, and prophylaxis of acute mountain sickness. Lancet 1976;2:1149–55. 62991 10.1016/s0140-6736(76)91677-9

[25] Yang SL Ibrahim NA Jenarun G Incidence and determinants of acute mountain sickness in mount Kinabalu, Malaysia. High Alt Med Biol 2020. 10.1089/ham.2020.0026 PMC7482124 32614265

[26] Cheng FY Jeng MJ Lin YC Incidence and severity of acute mountain sickness and associated symptoms in children trekking on Xue Mountain, Taiwan. PloS One 2017;12:e0183207. 28832689 10.1371/journal.pone.0183207 PMC5568320

[27] Basnyat B High altitude cerebral and pulmonary edema. Travel Med Infect Dis 2005;3:199–211. 17292039 10.1016/j.tmaid.2004.06.003

[28] Schilling M Irarrazaval S Ibuprofen versus acetazolamide for prevention of acute mountain sickness. Medwave 2020;20:e7733. 32604398 10.5867/medwave.2020.05.7732

